# In vivo neutralization of dendrotoxin-mediated neurotoxicity of black mamba venom by oligoclonal human IgG antibodies

**DOI:** 10.1038/s41467-018-06086-4

**Published:** 2018-10-02

**Authors:** Andreas H. Laustsen, Aneesh Karatt-Vellatt, Edward W. Masters, Ana Silvia Arias, Urska Pus, Cecilie Knudsen, Saioa Oscoz, Peter Slavny, Daniel T. Griffiths, Alice M. Luther, Rachael A. Leah, Majken Lindholm, Bruno Lomonte, José María Gutiérrez, John McCafferty

**Affiliations:** 1 Department of Biotechnology and Biomedicine, Technical University of Denmark, Søltofts Plads 224, DK-2800 Kongens Lyngby, Denmark; 2 IONTAS Ltd., Iconix Park, London Road, Pampisford, Cambridgeshire, CB22 3EG United Kingdom; 3 Instituto Clodomiro Picado, Facultad de Microbiología, Universidad de Costa Rica, San José, 11501-2060 Costa Rica

## Abstract

The black mamba (*Dendroaspis polylepis*) is one of the most feared snake species of the African savanna. It has a potent, fast-acting neurotoxic venom comprised of dendrotoxins and α-neurotoxins associated with high fatality in untreated victims. Current antivenoms are both scarce on the African continent and present a number of drawbacks as they are derived from the plasma of hyper-immunized large mammals. Here, we describe the development of an experimental recombinant antivenom by a combined toxicovenomics and phage display approach. The recombinant antivenom is based on a cocktail of fully human immunoglobulin G (IgG) monoclonal antibodies capable of neutralizing dendrotoxin-mediated neurotoxicity of black mamba whole venom in a rodent model. Our results show the potential use of fully human monoclonal IgGs against animal toxins and the first use of oligoclonal human IgG mixtures against experimental snakebite envenoming.

## Abstract (web summary)

Current anti-venoms against black mamba (*Dendroaspis polylepis*) bites are animal-derived and associated with several limitations. Here, Laustsen and colleagues develop an experimental recombinant anti-venom based on oligoclonal human IgG antibodies and establish its potential protective value in neutralizing dendrotoxin-mediated neurotoxicity using venom challenge in vivo models.

## Introduction

Snakebite envenoming exacts a death toll of 80–150,000 victims each year, leaves approximately four times as many maimed for life^1^, and has recently been recognized as a Neglected Tropical Disease by the World Health Organization (WHO)^2^. Antivenoms derived from the plasma of hyper-immunized animals remain the mainstay of snakebite envenoming therapy^3^. However, these present a range of drawbacks due to their relatively high cost and their heterologous nature that causes, in some patients, a number of side effects, such as serum sickness and early adverse reactions, which may include severe anaphylaxis^4–6^. Furthermore, it is estimated that only a fraction of the antibodies in most current antivenoms have a therapeutic value, as the majority of antibodies isolated from animal plasma are directed against antigens that are either unrelated to snake venom or related to venom components with negligible contribution to venom toxicity^5^. The implication follows that high amounts of antivenom protein may be needed to treat a snakebite, with heterologous protein loads reaching as much as 15 g/treatment for some antivenoms in severe envenoming cases^7^. Particularly, elapid antivenoms often have an unbalanced antibody content with relatively low amounts of antibodies against small neurotoxic venom components that have low immunogenicity, which often leads to low immune responses in production animals^8–10^. Despite the maturity of immunotherapy, there remains a need for cost-effective antivenoms with improved safety and efficacy^5^.

The notorious black mamba (*Dendroaspis polylepis*) from sub-Saharan Africa is a particularly dangerous species due to its size, defensive nature, and fast-acting neurotoxic venom. Life threatening clinical manifestations of *D. polylepis* envenoming include flaccid paralysis due to blockade of neuromuscular transmission resulting from inhibition of nicotinic acetylcholine receptors in the peripheral nervous system caused by α-neurotoxins (both short-chain and long-chain types) of the three-finger toxin superfamily^11^. In terms of abundance, however, the venom of *D. polylepis* is dominated by dendrotoxins, which are a unique type of neurotoxins that inhibit voltage-dependent potassium channels causing excitatory effects that result in involuntary muscle contractions^12^. In the venom, short neurotoxins, long neurotoxins, and dendrotoxins combine synergistically to provide *D. polylepis* with a potent neurotoxic bite^11^.

Recently, recombinant antivenoms based on oligoclonal mixtures of human antibodies have been proposed as a cost-competitive alternative to current antivenoms^13^. Additionally, such recombinant antivenoms may provide safer and more efficacious snakebite therapies due to their compatibility with the human immune system and the possibility of only including antibodies of therapeutic value, targeting medically relevant snake venom toxins, in the antivenom mixture. To discover such antibodies, phage display has been identified as a promising technology^14^ and has already yielded a number of neutralizing antibody fragments targeting venom toxins from snakes (reviewed in Laustsen et al. 2016)^5^. However, to the best of our knowledge, no fully human IgG antibody has been reported against any venom toxin from any multicellular organism, let alone a snake. Human IgGs have the benefits over antibody fragments of a prolonged half-life and different effector functions that depend on the Fc fragment. This may be of great therapeutic value for neutralization of systemically-acting toxins that leak from the bite site in victims over the course of days^15,16^. Here, we report the discovery of a suite of human IgGs that provide protection in vivo against dendrotoxins from the black mamba when administered by intracereberoventricular injection. This discovery approach combined toxicovenomics^17^, antibody phage display technology^18^, antibody engineering, mammalian cell expression, and whole venom in vivo neutralization studies in rodents. These results, thus, provide a proof of concept that oligoclonal mixtures of recombinant human IgG antibodies can be exploited to treat envenoming by the black mamba.

## Results

### Description and preparation of venom antigens (toxins)

*D. polylepis* venom was fractionated using RP-HPLC^11^, resolving the key dendrotoxins in four venom fractions (Dp5, Dp6, Dp7, and Dp8) that cannot be further resolved in quantitative yields with standardized techniques. While Dp8 contains almost pure dendrotoxin-1 (P00979 (https://www.uniprot.org/uniprot/P00979)), the venom fractions Dp5, Dp6, and Dp7 are ‘mixed fractions’ that contain similar amounts of at least one dendrotoxin and at least one type II α-neurotoxins. Previous proteomic studies have identified the toxin components of Dp5, Dp6, and Dp7 to contain the same dendrotoxin (a homolog of dendrotoxin-δ, P00982 (https://www.uniprot.org/uniprot/P00982), from the Eastern green mamba, *D. angusticeps*) and a type II α-neurotoxin (α-elapitoxin Dpp2c, P01397 (https://www.uniprot.org/uniprot/P01397))^11^.

### Phage display selection and screening of scFv binders

**Fig. 1:**
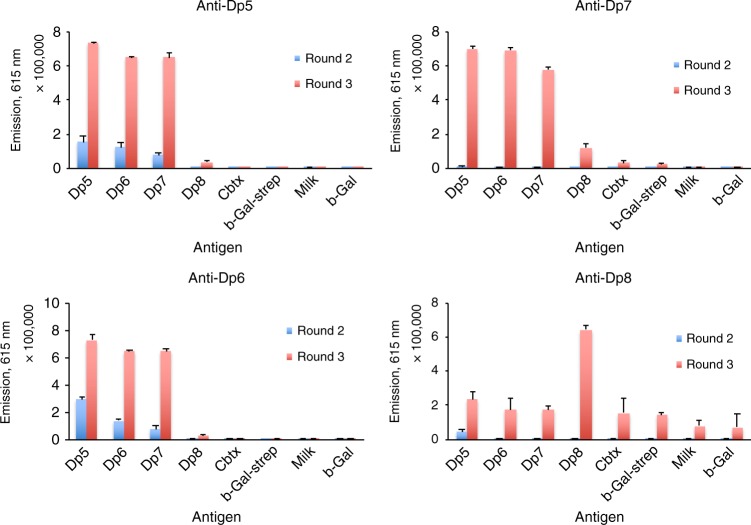
Polyclonal phage ELISA signals for the output phages for each selection. A significant increase in binding signal is observed from round 2 to 3 for all selections, and for selections against Dp5, Dp6, and Dp7, a high degree of cross-reactive binding exists for the phages against all three antigens (Dp5, Dp6, and Dp7). Negative control antigens: Cbtx α-cobratoxin, b-Gal β-galactosidase, Strep Streptavidin. All experiments were performed in triplicates on distinct samples. Error bars represent standard deviations

**Fig. 2:**
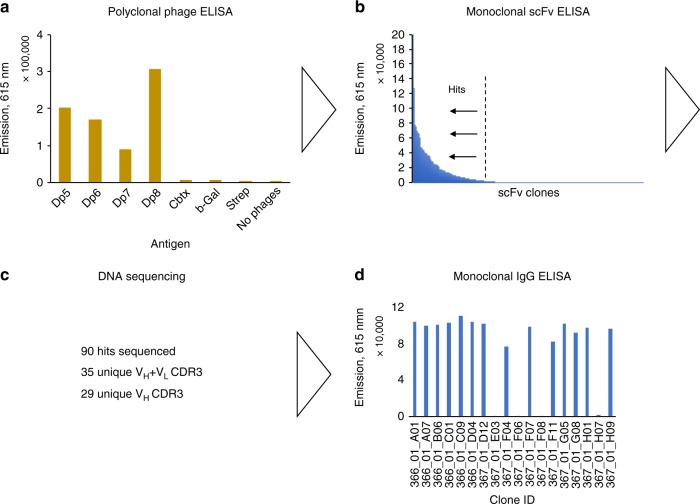
Schematic overview of selected results from the employed discovery process. Here, demonstrated for human IgGs against the antigen, Dp8. **a** Polyclonal phage ELISA signals against different venom fractions and negative control antigens (Cbtx α-cobratoxin, b-Gal β-galactosidase, Strep Streptavidin). **b** Monoclonal scFv ELISA signals against Dp8. **c** Summary of DNA sequencing results. Sequences are defined as unique based on V_H_ and V_L_ CDR3 sequences. **d** Monoclonal IgG ELISA signals for converted clones demonstrating retained binding for the majority of the clones upon conversion from the scFv format

Following three rounds of panning against selected venom fractions containing dendrotoxins, polyclonal ELISAs revealed that antibody binders had been enriched (Fig. 1). Antibody genes (in scFv format) were isolated from both the second and third panning rounds, sub-cloned into an scFv bacterial expression vector^19^, and 188 clones were picked and their antibody expressed^20^. Recombinant monoclonal antibodies were tested for binding to respective target antigens (see example with Dp8 as antigen Fig. 2). Using a cut-off score of 5000 fluorescence units (25 times above the background binding signal), the top binders (up to 94) were picked for each antigen for further characterization (DNA sequencing and affinity ranking). An Expression-Normalized Capture (ENC) assay was used to rank the antibody clones by affinity. In this assay, limiting amounts of anti-FLAG antibody were used to capture FLAG-tagged scFvs in the expression culture supernatant. Since the scFv expression level for each clone in culture supernatant is well above the capture capacity of the anti-FLAG antibody coated in each well, differences in antibody expression are normalized so that the binding signal better reflects differences in affinity between scFv and antigen.

### Conversion of scFvs to IgG format and characterization

A panel of unique scFv-formatted antibodies that yielded the highest binding signals in the ENC assay were selected for conversion to IgG format. Following expression by transient transfection in Expi293^TM^ cells, ELISA was used to confirm retention of target binding.

**Fig. 3:**
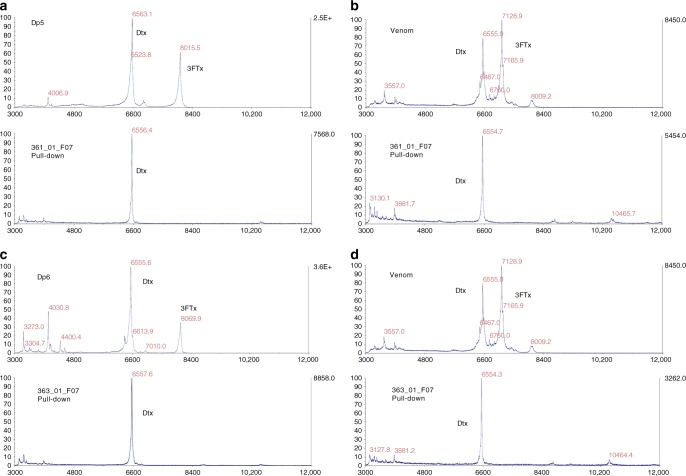
Example of MALDI-TOF MS analysis of antigen-antibody complex pull-down experiments. **a** IgG 361_01_F07 pull-down from venom fraction Dp5. **b** IgG 361_01_F07 pull-down from whole venom. **c** IgG 363_01_F07 pull-down from venom fraction Dp6. **d** IgG 363_01_F07 pull-down from whole venom. Dtx Dendrotoxin, 3FTx Three-finger toxin

Twenty-five IgG-formatted antibodies were produced and purified by protein A chromatography. Using a fluid-phase technique based on protein G-beads pull-down of antigen-antibody complexes, followed by acid dissociation and MALDI-TOF MS analysis (see example in Fig. 3), binding to a dendrotoxin homologous to dendrotoxin-δ^11^ in the venom fractions was confirmed for 4/4 IgGs against Dp5, 3/4 IgGs against Dp6, and 1/8 IgGs against Dp7 (Table 1). The Dp8 fraction was known to consist predominantly of dendrotoxin-1, with only minor traces of other toxins^11^. A pull-down experiment was performed for clone 367_01_H01, which confirmed that dendrotoxin-1 was indeed the target for this clone (Table 1).

**Table 1:** Overview of all MALDI-TOF MS pull-down experiments

IgG	Antigen	Mass(es) detected (venom fraction) (M + H)	Mass(es) detected (whole venom) (M + H)	Corresponds to
360_01_B12	Dp5	6555.1	*Ca. 6500 + 10465.9	“Dendrotoxin-δ“
360_01_C09	Dp5	6555.6	6554.8	“Dendrotoxin-δ“
361_01_F07	Dp5	6556.5	6554.6	“Dendrotoxin-δ”
361_01_G08	Dp5	6556.2	6555.8	“Dendrotoxin-δ“
362_01_A08	Dp6	6556.7	6554.5	“Dendrotoxin-δ“
362_01_D01	Dp6	6557.4	6554.2	“Dendrotoxin-δ“
363_01_F07	Dp6	6557.6	6554.3	“Dendrotoxin-δ“
363_01_G12	Dp6	8069.7	*Ca. 8000 + ca. 10400	Type II α-neurotoxin
364_01_A04	Dp7	8002.6 + 8067.0	8007.2 + 8068.2	Type II α-neurotoxin
364_01_A01	Dp7	8005.2 + 8024.8 + 8069.7	8008.2 + 8066.4 (+*ca. 10400)	Type II α-neurotoxin
364_01_B01	Dp7	8002.2 + 8065.9	8005.9 + 8067.3	Type II α-neurotoxin
364_01_C11	Dp7	8006.1 + 8066.9	N.t.	Type II α-neurotoxin
364_01_D03	Dp7	8005.5 + 8025.4	*(−)	Type II α-neurotoxin
364_01_D04	Dp7	8004.8 + 8069.3	*Ca. 8000	Type II α-neurotoxin
365_01_F03	Dp7	8005.4 + 8028.8 + 8068.5	*(−)	Type II α-neurotoxin
365_01_G06	Dp7	6560.3	6553.8	“Dendrotoxin-δ“
367_01_H01	Dp8	N.t.	7127.4	Dendrotoxin-1

^*^indicates a poor signal-to-noise ratio. N.t. not tested. (−) = could not be determined. M + H = determined molecular mass + proton. “Dendrotoxin-δ“ denotes a *D. polylepis* dendrotoxin homologous to the *D. angusticeps* dendrotoxin-δ. Instrumental error is within 0.02% of the observed mass values

### In vivo neutralization of dendrotoxins

In total, 24 out of 25 recombinant human IgGs targeting black mamba neurotoxins were tested in vivo. All IgGs were evaluated for neutralization of lethality by the intracerebroventricular (i.c.v.) route, where nine showed full (100%) protection against the venom fraction they were raised against (Tables 2 and 3). Even at the highest dose tested, seven IgGs failed to provide survival in the 24 h assay, although most of these IgGs showed prolonged survival time, as compared to controls, during the assay. Eight IgGs provided partial survival in the 24 h assay at one or more dose regimes (Tables 2 and 3).

**Table 2:** In vivo neutralization results for monoclonal IgG antibodies raised against Dp5, Dp6, and Dp7

	Venom fractions																
IgG ID	Dp5						Dp6							Dp7			
	0.5:1	0.75:1	1:1	2:1	3:1	4:1	0.5:1	0.75:1	1:1	2:1	3:1	4:1	6:1	2:1	3:1	4:1	6:1
360_01_B12				0/4	4/4	4/4						1/4				1/4	
360_01_C09				0/4	1/4	4/4						0/4				1/4	
361_01_F07	2/4	3/4	3/4	4/4	4/4	3/4						2/4				1/4	
361_01_G08	0/4	1/4	2/4	2/4	4/4	4/4						1/4				0/4	
362_01_A08		3/4	3/4	4/4	4/4	4/4			1/4	2/4	3/4	3/4				0/4	
362_01_D01		2/4	3/4	3/4	3/4	3/4			0/4	2/4	2/4	4/4				1/4	
363_01_F07		4/4	4/4	4/4	4/4	4/4	0/4	0/4	2/4	4/4	4/4	4/4				1/4	
363_01_G12												0/4	0/4				
364_01_A01														0/4	0/4*	0/4	0/4
364_01_A04						0/4						0/4			1/4	3/4	
364_01_B01															1/4*	1/4	1/4
364_01_C11																1/4	
364_01_D03															0/4*	0/4	0/4
364_01_D04															0/4*	1/4	0/4
365_01_G06		4/4	3/4	4/4	4/4	4/4				0/4	0/4	3/4		0/4	3/4	4/4	

Monoclonal IgGs were tested against individual venom fractions using i.c.v administration. Numbers indicate survival ratios at 24 h. * denotes that intravenous (i.v.) administration was used instead of i.c.v. administration. Ratios are provided as molar ratios between IgG:toxin.

The i.c.v. assay is particularly useful for assaying toxicity of dendrotoxins, as these neurotoxins are highly potent when administered i.c.v., but display lower toxicity by intravenous (i.v.) administration, requiring relatively high doses to induce lethality^11^. In contrast, α-neurotoxins are less potent when administered i.c.v. and are better assayed using the i.v. route of administration. Three of the four investigated venom fractions contain a mixture of dendrotoxins and type II α-neurotoxins^11^, and it was observed that only dendrotoxin-targeting IgGs were able to provide full survival in the i.c.v. assay (compare Table 1 with Tables 2 and 3). Clones 364_01_A01, 364_01_B01, 364_01_D03, and 364_01_D04 target the type II α-neurotoxin present in Dp7 and were therefore also assayed using the i.v. route. Unfortunately, these IgGs failed in providing full protection, although clone 364_01_B01 provided a low survival rate (1/4) when mice were challenged with 10.6 µg toxin pre-incubated with the IgG at a molar ratio of 3:1 (IgG:toxin). Similarly, clone 364_01_A01 provided significantly prolonged survival, although all challenged mice died between 12–18 h. The present findings thus demonstrate that effective dendrotoxin-targeting human IgGs were discovered, but more work (such as affinity maturation) may be needed to improve the discovered IgGs that target type II α-neurotoxins. Moreover, this work also demonstrates that the discovered human IgGs could neutralize the lethal effect of the target dendrotoxins present in more than one venom fraction (e.g., 363_01_F07 which completely neutralizes the dendrotoxins in Dp5 and Dp6 and provides some protection against Dp7) (Table 2). The mass spectrometry data (Table 1) suggests that, in this case, the observed neutralization ability is due to the presence of a key dendrotoxin (or very similar dendrotoxins) in more than one venom fraction.

**Fig. 4:**
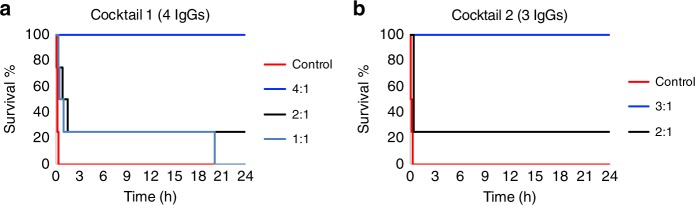
Kaplan-Meier survival curves for antibody cocktails. Here, shown for **a** Cocktail 1 and **b** Cocktail 2 at different molar ratios against black mamba whole venom administered i.c.v., demonstrating full protection at an IgG:toxin molar ratio of 4:1 for Cocktail 1 and 3:1 for Cocktail 2. Cocktail 1 contains the IgGs: 361_01_F07 (anti-Dp5), 363_01_F07 (anti-Dp6), 365_01_G06 (anti-Dp7), 367_01_H01 (anti-Dp8). Cocktail 2 contains the IgGs: 363_01_F07 (anti-Dp6), 365_01_G06 (anti-Dp7), 367_01_H01 (anti-Dp8). Each individual curve represents survival of a cohort of 4 animals

**Fig. 5:**
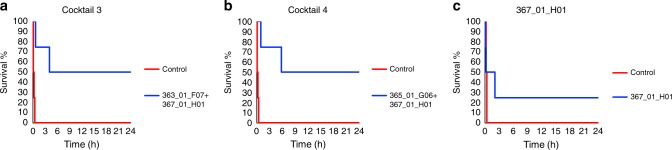
Kaplan–Meier survival curves for antibody cocktails. Here, shown for **a** Cocktail 3, **b** Cocktail 4, and **c** clone 367_01_H01 against black mamba whole venom administered i.c.v. at an IgG:toxin molar ratio of 3:1. Cocktail 3 contains the IgGs: 363_01_F07 (anti-Dp6) and 367_01_H01 (anti-Dp8). Cocktail 4 contains the IgGs: 365_01_G06 (anti-Dp7) and 367_01_H01 (anti-Dp8). Each individual curve represents survival of a cohort of 4 animals

To explore whether the dendrotoxin-mediated neurotoxicity of the whole venom could be completely abrogated using the discovered human IgGs, antibody cocktails were designed (Table 4) and evaluated against whole venom by the i.c.v. route. In all cases, the combination of antibodies to dendrotoxin-1 and the dendrotoxin-δ homolog was superior to anti-dendrotoxin-1 (367_01_H01) alone. Anti-dendrotoxin-1 (367_01_H01) was used along with different combinations of antibodies to dendrotoxin-δ. For example, Cocktail 2 included 363_01_F07 and 365_01-G06 whereas Cocktail 1 included these, as well as an additional antibody (361_01_F07) (Tables 3 and 4). Both cocktails successfully provided full protection against whole venom when injected via the i.c.v. route at a challenge dose of 1.5 µg of whole venom pre-incubated with the IgG cocktails at IgG:toxin molar ratios of 4:1 and 3:1 (Fig. 4). Omission of one or other of the anti-dendrotoxin-δ-homolog antibodies from Cocktail 2 yielded reduced protection. For example, neutralization tests against whole venom were performed i.c.v. using Cocktail 3 (where 365_01_G06 had been substituted with additional 363_01_F07), Cocktail 4 (where 363_01_F07 had been substituted with additional 365_01_G06) (Table 4), and 367_01_H01 alone. As seen in Fig. 5, none of these cocktails were able to provide equivalent protection at equimolar doses compared to Cocktail 2. Thus, Cocktail 2 represented the minimum cocktail providing full protection in this experiment.

**Table 3:** In vivo neutralization results for monoclonal IgG antibodies raised against Dp8 and oligoclonal IgG cocktails

	Venom fractions						
IgG ID	Dp8			Whole venom			
	2:1	4:1	6:1	1:1	2:1	3:1	4:1
366_01_A01	0/3	0/3	1/3				
366_01_B06	0/3	0/3	0/3				
366_01_C01	0/3	0/3	0/3				
366_01_C09	2/3	2/3	0/3				
367_01_F04	0/3	0/3	0/3				
367_01_F07	1/3	0/3	3/3				
367_01_F11	2/3	0/3	2/3				
367_01_H01	3/3	3/3	3/3			1/4	
367_01_H09	0/3	1/3	1/3				
Cocktail 1				0/4	1/4		4/4
Cocktail 2					1/4	4/4	
Cocktail 3						2/4	
Cocktail 4						2/4	

Monoclonal IgGs and IgG cocktails were tested against individual venom fractions and whole venom using i.c.v administration. Numbers indicate survival ratios at 24 h. Ratios are provided as molar ratios between IgG:toxin.

**Table 4:** Composition of the IgG cocktails

	anti-Dp5 361_01_F07	Anti-Dp6 363_01_F07	Anti-Dp7 365_01_G06	Anti-Dp8 367_01_H01
Cocktail 1	1	1	1	6
Cocktail 2		2	1	6
Cocktail 3		3		6
Cocktail 4			3	6

Numbers in table represent relative molar ratios between each IgG component

Finally, Cocktail 1 and 2 were tested against whole venom by the i.v. route to demonstrate that these cocktails were truly dendrotoxin-specific and that whole venom cannot be neutralized, when using the i.v. route, unless both dendrotoxins and α-neurotoxins in the venom are neutralized^21^. As expected, similar survival curves (rapid death upon injection) were observed for mice challenged with 25.8 µg of whole venom alone and mice challenged with a similar dose of whole venom pre-incubated with Cocktail 1 and 2 at molar ratios of 3:1 and 4:1 (IgG:toxin), respectively.

For comparative purposes, an equine-derived F(ab’)_2_ polyclonal antivenom was tested for its efficacy against the lethal effect of *D. polylepis* venom by the i.c.v. route. This antivenom had been previously shown to be highly effective in the neutralization of lethality of this venom by the i.v. route, with a Median Effective Dose of 5.25 mg venom neutralized per mL antivenom^11^, an observation that was confirmed in the present study. In contrast, when lethality was tested by the i.c.v. route, the antivenom failed to neutralize this venom even at a ratio of 0.33 mg venom per mL antivenom, as all mice receiving the mixture of venom and antivenom died, whereas control mice injected with antivenom alone survived.

## Discussion

The results presented here are the first report of the use of human IgG antibodies capable of neutralizing animal toxins in vivo. Moreover, with this report, we demonstrate that the dendrotoxin-mediated neurotoxicity of whole venom of the black mamba can be neutralized in an i.c.v. rodent model using carefully selected oligoclonal mixtures of monoclonal human IgGs. Our results further indicate that individual monoclonal dendrotoxin-targeting IgGs cannot achieve this alone, and it is likely to be essential to employ antibody mixtures that neutralize several key toxins of black mamba whole venom to achieve full protection against dendrotoxin-mediated neurotoxicity. Finally, through systematic testing, we determined a minimum IgG cocktail (Cocktail 2) containing three human IgGs that is capable of providing full protection against lethality in mice co-injected with lethal doses of whole venom by the i.c.v. route. The value of these antibodies is underscored by the fact that a polyvalent antivenom in clinical use, which is highly effective in the neutralization of *D. polylepis* venom when tested by the i.v. route, failed to neutralize venom toxicity when assays were performed by the i.c.v. route. Since dendrotoxins are the predominant neurotoxins upon i.c.v. injection, whereas α-neurotoxins predominantly mediate toxicity when using the i.v. route, our findings suggest that the equine polyvalent antivenom has limitations for the neutralization of dendrotoxins, hence reinforcing the potential of recombinant antibodies against this type of neurotoxin.

Three limitations exist for this work. (1) When using an i.c.v. model, dendrotoxins are the predominant toxic components, and neutralization of whole *D. polylepis* venom can be achieved by neutralizing these toxins only. When switching to an i.v. model, neutralization of α-neurotoxins is likely to be essential, as these are expected to be the main drivers of toxicity by this route. This speculation is supported by our finding that both IgG cocktails tested in this work could not provide survival against whole venom when i.v. administration was employed. (2) A model based on i.c.v. does not reflect a typical envenoming, but only demonstrates the ability of toxin-targeting IgGs to neutralize some neurotoxins. Thus, despite the usefulness of the i.c.v. route to assess the neutralization of dendrotoxins, the use of the i.v. route is recommended to test neutralization of α-neurotoxins and crude venoms. (3) Finally, although incubation and co-administration of venom and IgGs follows the recommended WHO guidelines^22^, such experiments fail to account for the impact of toxicokinetics and pharmacokinetics. It would be relevant to follow up on these observations with ‘rescue’ experiments, where IgGs are administered i.v. after a period of time following administration of whole venom i.m. or s.c., as this will better reflect a real-life envenoming situation.

Taken together, the data provided here constitutes a proof of concept for the use of oligoclonal mixtures of recombinant human IgGs against snakebite envenoming. The use of carefully selected human IgG antibodies holds the promise of delivering safer and more effective treatments against snakebite envenoming due to the compatibility with the human immune system and the possibility of only including antibodies of therapeutic value in a recombinant antivenom^5,6^. Moreover, as cost-efficacy remains one of the main challenges of delivering biotherapeutics to patients in developing countries^1^, the use of oligoclonal recombinantly expressed human IgGs has been theorized to be cost-competitive with current plasma-derived antivenoms^13^. These results bring hope that recombinant antivenoms based on fully human IgG antibodies may in the future be available for the therapy against snakebite envenomings.

## Methods

### Venom fractionation

Pooled *D. polylepis* venom from several specimens originating from Kenya was obtained in lyophilized form from Latoxan SAS, France. Venom fractions Dp5, Dp6, Dp7, and Dp8 containing dendrotoxins from *D. polylepis* were isolated from crude venom by RP-HPLC (Agilent 1200) on a C_18_ column (250 × 4.6 mm, 5 μm particle; Teknokroma). Elution was carried out at 1 mL/min using Solution A (water, containing 0.1% TFA) and a gradient towards solution B (acetonitrile, containing 0.1% TFA): 0% B for 5 min, 0–15% B over 10 min, 15–45% B over 60 min, 45–70% B over 10 min, and 70% B over 9 min. Fractions were collected manually and dried in a vacuum centrifuge^11^.

### Biotinylation and MS analysis of toxins

Venom fractions were dissolved in phosphate buffered saline (PBS, Dulbecco’s Phosphate Buffered Saline; Sigma-Aldrich) to yield concentrations of 0.85–6.39 μg/μL. Biotin linked to *N*-hydroxysuccinimide (NHS) via a PEG_4_-linker (EZ-Link™ NHS-PEG_4_-Biotin, No-Weigh™ Format, Thermo Scientific, 21329) was added to the toxin solutions at molar ratios of 1:1 to 1:2 (toxin: biotinylation reagent) and left at room temperature for 30 min. Buffer exchange columns (Vivacon 500, Sartorius, 2000 Da Molecular Weight Cut-Off) were employed for purification of the biotinylated toxins using three washes of 500 μL PBS and an elution volume of 150 μL PBS. Protein concentrations were determined based on individually calculated extinction coefficients (http://web.expasy.org/protparam/) and absorbances measured on a BMG labtech PHERAStar Fluorescence Spectrophotometer. The extent of biotinylation was analyzed by MALDI-TOF in a Proteomics Analyzer 4800 Plus mass spectrometer (Applied Biosystems) to ensure that over-biotinylation had not taken place^23^.

### Phage display selection and primary screening

For phage display selection, the IONTAS phage display library was employed. This library is a human antibody phage display library of 4 × 10^10^ clones, with antibodies in the form of single chain variable fragments (scFvs), which was constructed from B lymphocytes collected from 43 non-immunized human donors^24^. Selection of scFv binders from the IONTAS phage display library and primary TRF assay screening for dendrotoxin binders were performed as described elsewhere^24,25^. Briefly, selected antibodies were sub-cloned from the phage display vector using *Nco* I and *Not* I restriction endonuclease sites into a vector for expression of soluble scFvs^19^ and transformed into *E. coli* strain BL21 (DE3) (New England Biolabs). Individual scFv clones (up to 188 scFv clones against each target) were picked, expressed in 96-well format, and scFv-containing supernatants tested for binding to their corresponding venom fraction targets (1–5 μg/mL) indirectly immobilized on streptavidin (10 μg/mL) coated MaxiSorp plates using the DELFIA system^25^. For binding detection, 1 in 1500 dilution of anti-FLAG M2 (Sigma, F1804) conjugated with Europium (custom labeled by Perkin Elmers) was used. Ninety-four binders against each target were cherry-picked and sequenced (Eurofin Genomics sequencing service) using S10b primer (GGCTTTGTTAGCAGCCGGATCTCA). The antibody framework and CDR regions were annotated and analyzed to identify unique clones.

### Expression-normalized capture (ENC) assay

For the ENC assays, black MaxiSorp plates (Nunc) were coated overnight with anti-FLAG M2 antibody (Sigma, 2.5 μg/ml in PBS, 50 μL per well). After blocking with 2% M-PBS (skim milk in PBS), washing with PBS, and addition of 30 μL of 6% M-PBS to each well, 30 μL of individual auto-induction culture supernatants^20^ containing expressed scFv was added for each scFv to the assay plate. Plates were washed three times with PBS-T (PBS, 0.1% Tween-20) and three times with PBS. Binding of biotinylated antigen (tested using both 2.5 nM and 25 nM of each antigen in 2% M-PBS, 50 μL per well for 1 h) was detected using Europium-labeled Streptavidin (Perkin Elmer, 1244-360, 1 μg/mL in PBS-M, 50 μL per well for 30 min).

### IgG expression and purification

**Fig. 6:**
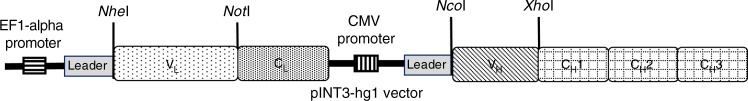
Schematic representation of the genetic elements present in the pINT3-hg1 vector

V_H_ and V_L_ genes of 30 antibodies that showed highest binding signal in the ENC assay were sub-cloned into a dual promoter mammalian expression vector. The pINT3-hg1 vector (Fig. 6) has a dual promoter expression cassette, in which the heavy chain expression is controlled by the cytomegalovirus (CMV) promoter, and the light chain expression is driven by elongation factor-1 alpha (EF1-alpha) promoter. An alternative vector (pINT54-hg1) with a dual CMV promoter driving both heavy and light chain expression was also available and used in some cases. V_H_ chains of the selected antibodies were amplified from the pSANG10 scFv vector using primers pSang10_pelB (CGCTGCCCAGCCGGCCATGG) and HLINK3_R (CTGAACCGCCTCCACCACTCGA), and the V_L_ chains were amplified using primers LLINK2_F (CTCTGGCGGTGGCGCTAGC) and 2097_R (GATGGTGATGATGATGTGCGGATGCG). Amplified V_H_ genes were digested with *Nco* I and *Xho* I restriction endonucleases, and the V_L_ genes were digested with *Nhe* I and *Not* I restriction endonucleases. Digested V_H_ and V_L_ gene fragments were ligated into the pINT3-Hg1 or pINT54 hg1 plasmids (digested with *Nhe* I and *Xho* I restriction endonucleases) along with a stuffer fragment (digested with *Nco* I and *Not* I restriction endonucleases) encoding the constant light chain region (C_L_) and the CMV promoter as a four-part ligation using T4 DNA ligase (Roche, 10481220001).

In order to express the IgG antibodies in mammalian cells, transfection quality DNA was prepared using Plasmid Plus Kit (Qiagen, 12945). A total of 180 μg of the DNA was transfected into 180 mL of Expi293^TM^ cells (Thermo Fisher) using ExpiFectamine^TM^ 293 Transfection Kit (Thermo Fisher, A14525) according to manufacturer’s instructions. Cells were harvested after 6 days of incubation at 37 °C, 5% CO_2_, 130 rpm on a 25 mm orbital shaker. Antibodies were purified from the culture supernatants by protein A chromatography using the Äkta Pure system (GE Healthcare). The initial purification was performed using HiTrap MabSelect SuRe 5 mL column (GE Healthcare, 11-0034-94), using 0.1 M Citrate buffer (pH 3.0) for elution. Eluted proteins were neutralized with half the volume of 1 M Tris (pH 8.0) and dialyzed twice against 4 L of 2× PBS at 4 °C using GeBAflex Maxi dialysis tubes (Generon, D035). Dialyzed proteins were concentrated using Amicon ultra centrifugation filters (Merck Millipore, UFC905024) according to manufacturer’s instructions. The functionality of purified IgGs was confirmed by a TRF binding assay. In this assay, IgG binding to biotinylated venom fractions immobilized on streptavidin coated Nunc MaxiSorp plates was detected using an anti-human antibody conjugated with Europium (Perkin Elmers, 1244-330, 1 in 1000 dilution).

### Identification of target toxin for IgGs

Each monoclonal IgG was mixed, at a 4:1 molar ratio (IgG:toxin), with 0.5 µg of the venom fraction which it was selected against, or with whole venom, and added to a 20 µL slurry of protein G-agarose beads (Sigma P7700), in 50 µL of 0.05 M Tris, 0.75 M KCl, pH 7.0 buffer containing 2% bovine serum albumin. The mixtures were incubated for 30 min at room temperature in a Thermomixer (Eppendorf) at 700 rpm. After centrifugation for 15 s at 5000×*g*, the beads were washed twice with 200 µL of the same buffer, twice with 200 µL of PBS, and finally twice with 200 µL of deionized water. After removing the supernatant from the last wash, the beads were resuspended in 20 µL of a saturated solution of α-cyano-**4**-hydroxycinnamic acid in 50% acetonitrile and 50% water, containing 0.1% trifluoroacetic acid and 1 mg/mL of monobasic ammonium phosphate. The samples were mixed by vortexing for a few seconds, centrifuged for 15 s, and 1 µL of the supernatant was spotted onto an Opti-TOF 384 plate, dried, and analyzed by MALDI-TOF on a 4800-Plus Proteomics Analyzer (Applied Biosystems). TOF spectra were acquired in linear positive mode, using 500 shots at a laser intensity of 4200. Identification of toxin family was achieved by comparing the obtained masses with previously reported values^26^.

### Animals

In vivo assays were conducted in CD-1 mice (18–20 g) of both sexes, supplied by Instituto Clodomiro Picado, following protocols approved by the Institutional Committee for the Use and Care of Animals (CICUA), University of Costa Rica. Mice were housed in cages of various sizes for groups of 4–12, and were provided food and water ad libitum.

### Neutralization studies by intracerebroventricular injection

The neutralization activity of individual IgGs and IgG cocktails against both dendrotoxins and whole venom was tested by intracerebroventricular (i.c.v.) injection in groups of three to four mice (18–20 g body weight, both sexes used) using different doses of each venom fraction and whole venom (0.5–1.5 µg per mouse) and different toxin:IgG molar ratios (1:0.5, 1:0.75, 1:1, 1:2, 1:3, 1:4, and 1:6). Venom and venom fraction doses were selected as to ensure 100% mortality. IgGs and venom fractions/whole venom were mixed, and solutions were incubated (30 min either at room temperature or at 37 °C). After incubation, mice were injected with a volume of 5–33 µL (dependent on IgG stock concentration). Irrelevant-specificity control mice were injected with venom fractions/whole venom and anti-lysozyme IgG dissolved in phosphate-buffered saline (PBS; 0.12 M NaCl, 0.04 M sodium phosphate buffer, pH 7.2), while vehicle-control mice were injected with venom fractions/whole venom in PBS. Time of death was recorded, and Kaplan–Meier curves were used to represent mouse survival. For comparative purposes, the SAIMR polyvalent antivenom (South African Vaccine Producers, batch BC02645) was tested in a similar fashion. This antivenom is an F(ab’)_2_ preparation obtained from the plasma of horses immunized with a mixture of the venoms of ten snake species, including *D. polylepis*^27^. Various dilutions of this antivenom were incubated with a fixed concentration of *D. polylepis* venom. Controls included venom alone and antivenom alone. After incubation, aliquots of 10 µL, containing 1.5 µg venom, were injected i.c.v., as described, and deaths were recorded. In addition, antivenom neutralization of lethality of *D. polylepis* venom was tested by the i.v. route, as previously described^11^.

### Neutralization studies by intravenous injection

The neutralization activity of the IgGs against three-finger toxins and whole venom was tested by intravenous (i.v.) injection in groups of four mice (18–20 g body weight), using a challenge dose of 20.1 µg for venom fraction Dp6, 10.6 µg for venom fraction Dp7, and 25.8 µg of whole venom, and a toxin:IgG molar ratio of 1:3. Venom and venom fraction doses were selected as to ensure 100% mortality. IgGs and venom fractions/whole venom were mixed and incubated (30 min at room temperature). Then, aliquots of the mixtures were injected in the caudal vein, using an injection volume of 100–300 µL. Irrelevant-specificity control mice were injected with venom fractions/whole venom and anti-lysozyme IgG dissolved in PBS, while vehicle-control mice were injected with venom fractions/whole venom in PBS. Time of death was recorded, and Kaplan–Meier curves were used to represent mouse survival.

## Data Availability

The data that supports the findings of this study are available from the corresponding authors upon reasonable request. Figures  1, 2, 3, 4, and 5 have associated raw data.
