# Publisher Correction: In vivo neutralization of dendrotoxin-mediated neurotoxicity of black mamba venom by oligoclonal human IgG antibodies

**DOI:** 10.1038/s41467-018-07480-8

**Published:** 2018-11-20

**Authors:** Andreas H. Laustsen, Aneesh Karatt-Vellatt, Edward W. Masters, Ana Silvia Arias, Urska Pus, Cecilie Knudsen, Saioa Oscoz, Peter Slavny, Daniel T. Griffiths, Alice M. Luther, Rachael A. Leah, Majken Lindholm, Bruno Lomonte, José María Gutiérrez, John McCafferty

**Affiliations:** 1 Department of Biotechnology and Biomedicine, Technical University of Denmark, Søltofts Plads 224, DK-2800 Kongens Lyngby, Denmark; 2 IONTAS Ltd., Iconix Park, London Road, Pampisford, Cambridgeshire, CB22 3EG United Kingdom; 3 Instituto Clodomiro Picado, Facultad de Microbiología, Universidad de Costa Rica, San José, 11501-2060 Costa Rica

Correction to: *Nature Communications* 10.1038/s41467-018-06086-4; published online 2 October 2018

In the original version of this article, the sixth sentence of the first paragraph of the Introduction incorrectly read ‘Particularly, elapid antivenoms often have an unbalanced antibody content with relatively low amounts of antibodies against small neurotoxic venom components that have low immunogenicity, which often leads to low immune cgqtns in production animals^8–10^’. The correct version states ‘responses’ instead of ‘cgqtns’. This has been corrected in both the PDF and HTML versions of the article.

